# Phytoplankton Growth and Microzooplankton Grazing in the Subtropical Northeast Atlantic

**DOI:** 10.1371/journal.pone.0069159

**Published:** 2013-07-23

**Authors:** Carlos Cáceres, Fernando González Taboada, Juan Höfer, Ricardo Anadón

**Affiliations:** Departamento de Biología de Organismos y Sistemas, Universidad de Oviedo, Oviedo, Asturias, Spain; University of Delaware, United States of America

## Abstract

Dilution experiments were performed to estimate phytoplankton growth and microzooplankton grazing rates during two Lagrangian surveys in inner and eastern locations of the Eastern North Atlantic Subtropical Gyre province (NAST-E). Our design included two phytoplankton size fractions (0.2–5 µm and >5 µm) and five depths, allowing us to characterize differences in growth and grazing rates between size fractions and depths, as well as to estimate vertically integrated measurements. Phytoplankton growth rates were high (0.11–1.60 d^−1^), especially in the case of the large fraction. Grazing rates were also high (0.15–1.29 d^−1^), suggesting high turnover rates within the phytoplankton community. The integrated balances between phytoplankton growth and grazing losses were close to zero, although deviations were detected at several depths. Also, O_2_ supersaturation was observed up to 110 m depth during both Lagrangian surveys. These results add up to increased evidence indicating an autotrophic metabolic balance in oceanic subtropical gyres.

## Introduction

Oligotrophic subtropical oceans cover around 40% of the Earth´s surface and are currently expanding [1]. Nutrient concentrations are very low during most of the year mainly as a consequence of phytoplankton activity and vertical stratification [2]. For this reason, phytoplankton biomass is typically lower than in other marine environments, and there is a higher contribution of picophytoplankton to total phytoplankton biomass [3]. However, these properties do not necessarily mean low phytoplankton growth rates, or low primary production: subtropical gyres resemble desserts in their low biomass, but regarding their growth rates they could be more similar to tropical forests [4].

There are a wide range of phytoplankton growth rate estimates (from <0.1 d^−1^, e.g.[5]; to more than 1 d^−1^, e.g.[4]), which surely arises from spatiotemporal heterogeneity [5], but maybe also from the lack of agreement between different measurement methods [6], [7]. Hence, phytoplankton growth rates derived from primary production estimates based on the ^14^C method [8] have frequently resulted in values near the lower range. In oligotrophic subtropical environments, high grazing rates [9], together with the release of dissolved organic carbon compounds [10], resulting in isotope cycling, might explain apparent low rates obtained with the ^14^C method [11]. This is not a trivial matter since the magnitude of phytoplankton growth rates is a key feature to understand the functioning of these ecosystems and their role in biogeochemical cycles.

Oligotrophic subtropical gyres could sustain high phytoplankton growth rates if nutrient utilization by phytoplankton was coupled to nutrient regeneration [12]. In this case, a top-down regulation of the phytoplankton community would prevail. Herbivores, mainly microzooplankton and nanozooplankton, would play an important role in maintaining high growth rates by controlling producerś biomass [13], avoiding severe competition for nutrients, and by taking an active part in nutrient regeneration [14]. On the contrary, without herbivory, low phytoplankton growth rates would result due to severe nutrient limitation. In this case a bottom-up regulation of the phytoplankton community would prevail. Also, because the contribution of the biological pump [15] to net carbon sequestration depends on the balance between primary production and respiration, the role of subtropical gyres in atmospheric CO_2_ regulation depends upon how the ecosystem functions, which is directly impacted by microzooplankton grazing activities.

The objective of this study was to assess phytoplankton growth rates and microzooplankton grazing rates in order to clarify the functioning of the microbial food web in the Northeast Atlantic subtropical gyre. To this purpose, we conducted a series of dilution experiments [16] during two Lagrangian surveys in the Eastern North Atlantic Subtropical Gyre (NAST-E province) [17]. In contrast to previous studies in the North East Atlantic [18], [19], [20], we measured growth and mortality rates of phytoplankton at different depths in the water column down to the Deep Chlorophyll Maximum (DCM), allowing us to characterize vertical variation and to estimate vertically integrated measurements. Lagrangian surveys were conducted near the center and at the eastern boundary of the North East Atlantic subtropical gyre, providing two ecologically contrasting scenarios encompassing the range of conditions found in this part of the Atlantic Ocean. Finally, we considered two different size fractions of phytoplankton, which allowed us to study potential differences in growth and grazing rates within the phytoplankton community.

## Materials and Methods

### Study Area and Survey

**Figure 1:**
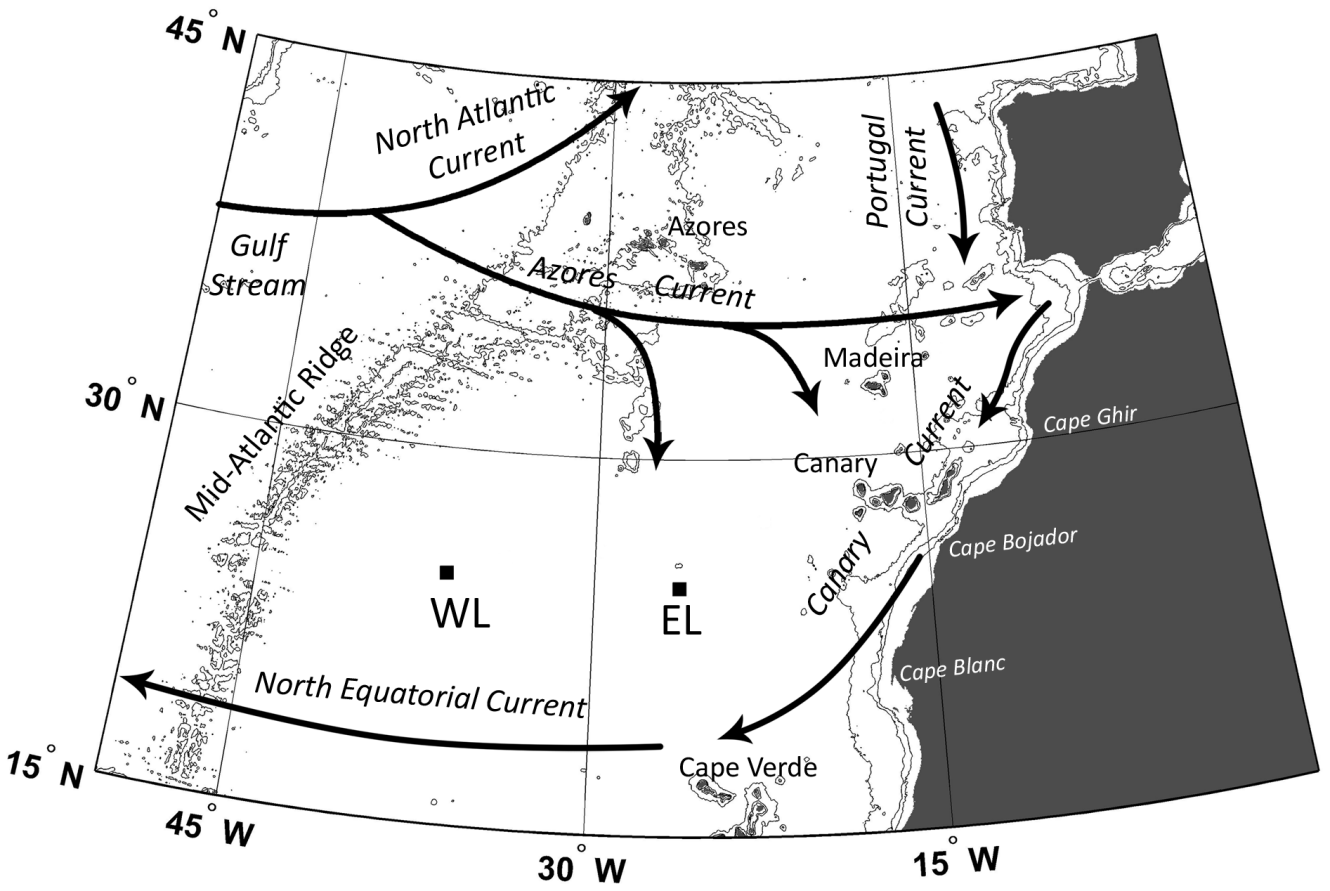
Map showing the study area and the location of the surveys. Two Lagrangian surveys were conducted during the CARPOS cruise near the center of the subtropical gyre, *i.e.* the West Lagrangian (WL), and near the eastern boundary, *i.e.* the East Lagrangian (EL).

The study was conducted as part of the CARPOS project (*Plankton and CARbon fluxes in Subtropical Oligotrophic environments: a Lagrangian approach*) aboard the RV ‘Hespérides’. Dilution experiments were carried out in the context of two Lagrangian surveys located around 25° N, 36° W (WL) and 25° N, 26° W (EL) (Fig. 1), within the NAST-E province. Experiments during WL were performed between October 25^th^ and 30^th^, 2006, while experiments during EL were conducted between November 15^th^ and 20^th^, 2006. Experiments in each Lagrangian survey were conducted during five consecutive days. Only one experiment was performed at each depth each day. The Lagrangian survey presents some advantages including the possibility of working in the same water body for several days, which allowed us to perform dilution experiments at several depths over consecutive days. The water body was tracked with a buoy joined to a drogue installed at 25 m depth. We obtained relative current velocities by using an Acoustic Doppler Current Profiler (ADCP) installed at the buoýs line, allowing us to estimate the deviation of the buoy with respect to the tracked water body (see Aranguren et al. [21] for further details).

### Water Column Properties

Vertical distributions of temperature, salinity, fluorescence, dissolved oxygen concentration (mg O_2_ L^–1^) and the percentage of oxygen saturation (O_2_ sat_,_ %) were obtained using a SBE-19 CTD, equipped with a SeaPoint fluorometer and a SeaBird SBE-43 oxygen meter. These variables were recorded between three and seven times per day. Water samples were obtained with a rosette equipped with 24 Niskin bottles of 12 L. Winkleŕs method was employed to calibrate the SBE-43 oxygen sensor (*R^2^* = 0.96). The depth of the photic layer (depth at which photosynthetic active radiation was 1% of the surface irradiance) was determined *in situ* from radiometer data. Nutrient analyses (NO_3_^–^, NO_2_^–^ and PO_4_^–^) were carried out from water collected between one and six times per day in polystyrene tubes, which were immediately frozen and preserved at −80°C for analysis using a Technicon AAII autoanalyser [22]. Only the data obtained on the days when dilution experiments were performed were retained for analysis.

### Plankton Abundance

Size-fractionated Chl *a* concentrations (mg Chl *a* m^–3^) were determined from initial samples of the dilution experiments, which were performed at 10, 30, 50 and 80 m depth, and at the DCM. Only one depth was sampled each day, corresponding to the depth of the dilution experiment for that day. We processed two 1000 ml samples from each depth. Samples were sequentially filtered through 5 µm and 0.2 µm pore diameter polycarbonate filters, which were arranged in line filter funnels. The filters were frozen and stored 24 h in dark. They were subsequently submerged in 90% acetone for 8–12 h. Chl *a* concentration was determined using the non-acidification technique [23] with a Turner Designs (TD-700) fluorometer calibrated with pure Chl *a*. From the two samples measured at each depth, we estimated the mean and the standard deviation (S.D.) of the Chl *a* concentration. We used those mean Chl *a* estimates to calculate the integrated Chl *a* up to 125 m depth by trapezoidal integration. Total Chl *a* concentration at each depth was determined by adding the two size-fractionated measurements.

Approximated carbon biomass was derived from size-fractionated Chl *a* data applying the C: Chl *a* ratios presented by Marañón et al. [24] for the North Atlantic Subtropical gyre: they were 103 at the upper mixed layer (UML) and 21 at the deep chlorophyll maximum (DCM) for the <2 µm phytoplankton fraction. Values for >2 µm phytoplankton were 247 at the UML and 60 at the DCM. C: Chl *a* ratios for phytoplankton <2 µm were used for phytoplankton <5 µm, while values for algae >2 µm were used for the algae >5 µm. Note that this is a conservative approach since C: Chl *a* ratios usually increase with the size of phytoplankton. Ratios for the UML were used from the surface up to the beginning of the DCM layer, defined as the depth where Chl *a* concentration was half of the DCM. The DCM was determined after examining SeaPoint fluorometer profiles. Finally, the relative abundance of diatoms, dinoflagellates, ciliates, radiolarians and copepod nauplii was estimated during EL at the same depths in which dilution experiments were performed. Samples (2.0 L) were processed using a FlowCam [25] configured in the autoImaging mode, with a 100 µm flow cell and a 10x objective.

The picophytoplankton community in EL was also analyzed by flow cytometry (FCM). Samples were fixed with a 1% paraformaldehyde plus 0.05% glutaraldehyde solution and stored at −80°C until analysis. A FacScan flow cytometer (Becton, Dickinson and Company) was used. Phytoplankton were grouped and enumerated according to side-scattered light (SSC), orange fluorescence (FL2, 585±21 nm) and red fluorescence signal (FL3, >650 nm). Samples were run at a flow rate between 38.6 and 43.2 µL min^−1^. Three groups were identified: *Prochlorococcus*, *Synechococcus* and picoeukaryotes. Cell abundances (mean ± S.D.) were obtained from the four initial undiluted samples analyzed at each depth (two from carboys with nutrient addition and another two from carboys without nutrient addition). We estimated the amount of biomass (carbon) in each cell detected by the flow cytometer using the conversion factors reported by Zubkov et al. [26] for *Prochlorococcus* (32 fg C cell^−1^) and *Synechococcus* (103 fg C cell^−1^), and Zubkov et al. [27] for picoeukaryotes (1.5 pg C cell^−1^). We integrated these average biomass values from each experimental depth over all depths sampled down to 125 m in each Lagrangian survey to estimate the integrated C biomass of each picoplankton group.

### The Dilution Method

The dilution method provides estimates of phytoplankton growth rate (*µ, d* ^−*1*^) and phytoplankton mortality rate (*m, d* ^−*1*^). The basis of the method is to uncouple phytoplankton growth rate from microzooplankton grazing by the addition of filtered seawater [16].The addition of filtered seawater dilutes the sample, reducing the encounter rates between phytoplankton and grazers and consequently phytoplankton grazing mortality (*m*) in an amount assumed to be linearly related to the dilution factor (*f*). Then, linear regression analysis of phytoplankton apparent growth rate (*r*) against *f* yields a slope and an intercept which corresponds to the rates of microzooplankton grazing and phytoplankton growth (*μ*), respectively.
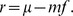



The balance between phytoplankton production and consumption is estimated as the difference between *μ* and *m* (i.e., *μ-m* balance, *d* ^−*1*^). It can be expressed in relative values, as the percentage of production grazed (% pNPP), dividing *m* by *μ.*

Apparent phytoplankton growth rate is estimated assuming an exponential growth model during the incubation:
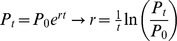



where *P_0_* and *P_t_* are observed population abundance (*Prochlorococcus* cells ml^−1^) or biomass (mg C m^−3^, calculated from size fractionated Chl *a* measurements) at initial and final times, respectively.

Different nutrient availabilities along dilution treatments could make *μ* change with the dilution, and cause non-linearities in the dilution relationship to occur. This problem can be avoided by providing an appropriate mixture of inorganic nutrients [16]. We followed this recommendation in the first experiments, conducted at 80 m depth and DCM in WL. Phytoplankton apparent growth rates were compared between treatments with added nutrients (*r_n_*) and no added nutrients (*r_0_*). Assuming that mortality rates were unaffected by the nutrient additions, these treatments allowed the calculation of growth rates (*μ_0_*) in natural seawater [28]:
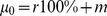
where *r_100%_* is the net growth rate observed in non-enriched undiluted containers. However, in those two first experiments, we did not find any effect of nutrient addition on phytoplankton net growth rates in the undiluted containers, which contain the higher biomass and possibilities of nutrient effect. Thus, in the rest of experiments (except at 50 m depth) nutrients were only added to two undiluted containers to check possible effects.

Particulate net primary production (pNPP, mg C m^−3^ d^−1^) and grazing losses (G, mg C m^−3^ d^−1^) were estimated using C: Chl *a* ratios (see the subsection *Plankton abundance*) and the equations proposed by Landry et al. [29] based on Frost [30]:
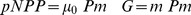


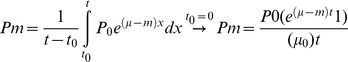
where *P_m_* is the mean concentration of phytoplankton (mg C m^−3^) during each experiment.

We estimated net changes of Chl *a* in the sea at the same depths and times of the dilution experiments (less than 2h of difference) to check their relationship with *μ-m* balances (considering both size fractions together) obtained from dilution experiments. To do that, we used CTD fluorescence data near the initial and final times of each dilution experiment and assumed an exponential phytoplankton growth model. Data below the UML were not included in the analysis to avoid the influence of processes like vertical displacement of the DCM that might affect net changes in Chl *a* concentration and hamper the detection of any relationship with *μ-m* balance.

**Figure 2:**
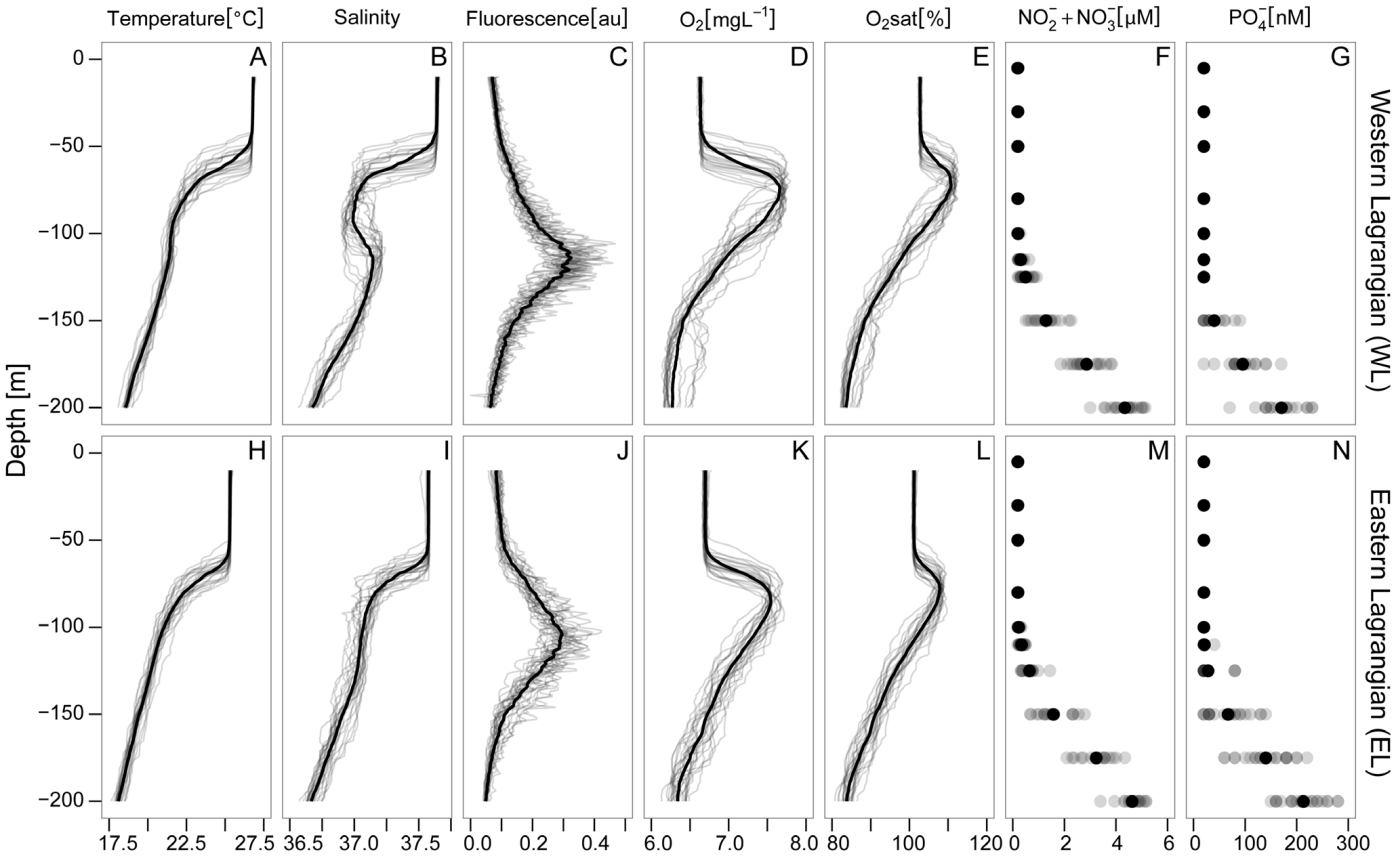
Properties of the water column during dilution experiments. Vertical profiles of temperature (A, H), salinity (B, I), fluorescence (C, J), oxygen (D, K), O_2_ saturation (E, L), nitrate plus nitrite (F, M) and phosphate (G, N) in the upper 200 m during dilution experiments in WL (top) and EL (bottom). Each grey line represents a different profile. Grey points represent nutrient concentrations. An additive transparency factor was applied to appreciate the agreement between values, being color intensity proportional to the number of overlapped lines or points, respectively. Black solid lines and points stand for average values.

**Figure 3:**
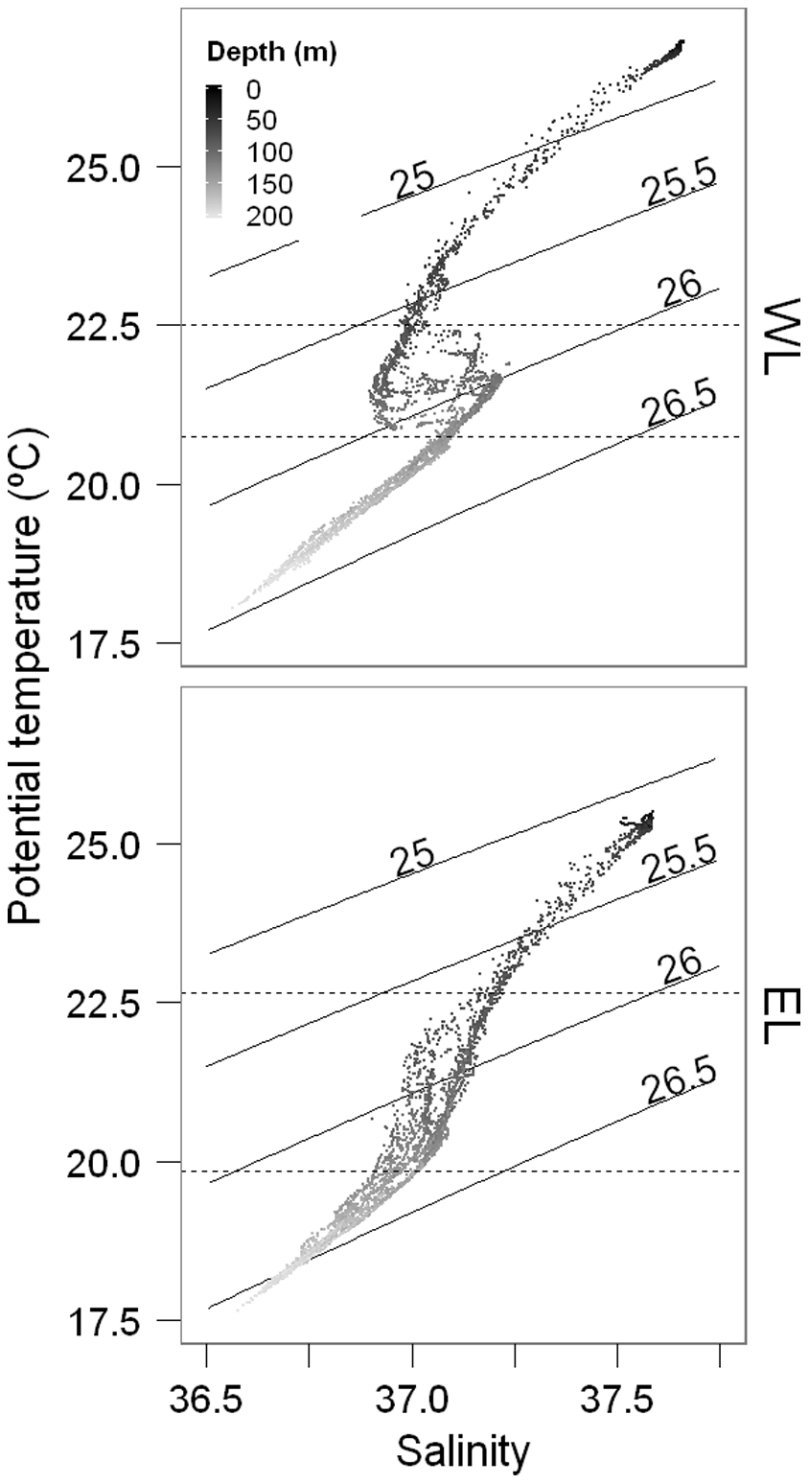
θ*-S* diagrams during dilution experiments. θ*-S* plots obtained from all the potential temperature (θ) and *S* measurements conducted in the upper 200 m during dilution experiments in WL (top) and EL (bottom). The color intensity of the dots indicates the depth associated to each θ*-S* pair. Black lines are isopycnals. Numbers above them point out sigma-theta values (σ_θ_ = potential density −1000 Kg m^−3^). Dotted lines are isotherms between which θ*-S* plots dispersion was higher.

Despite conducting the dilution experiments at each depth on consecutive days within a Lagrangian transect, we integrated the rates vertically to obtain a synoptic view of the ecosystem. We ignored in this way potential changes between days which we considered secondary with respect to changes through the water column and between the locations of each Lagrangian survey. This view was reinforced by the low temporal variation in physical-chemical conditions and the absence of abrupt temporal changes in Chl *a* concentrations (Fig. 2, see also [21]). Hence, θ-*S* plots from different vertical profiles conducted during the days in which dilution experiments were performed overlapped quite well, except at the base of the thermocline, suggesting a good tracking of the water body (Fig. 3). Integrals (0–125 m) of pNPP, G, and pNPP-G were determined using the trapezoidal method. Then, these integrals were divided by phytoplankton carbon biomass, resulting in integrated values for *μ*, *m*, and *μ-m*, which has the effect of accounting for differences in biomass between size fractions and depths.

### Experimental Setup, Sampling and Analysis Procedure

Water for the dilution experiments was collected at five depths: 10, 30, 50, 80 m and at the DCM (115 m in WL and 110 m in EL). Each day, water from one depth was collected at 4∶30–5∶00 h GMT, always before dawn, using two 30 L Niskin bottles. Lights onboard were turned off during sampling, except for minimal safety requirements. Carboys, containers, capsule filters and auxiliary pipes were stored in 10% HCl-Milli-Q water between experiments, and rinsed sequentially with Milli-Q water and 0.2 µm filtered seawater immediately before use. Capsules were changed every four experiments. We checked that water filtration did not increase the inorganic nitrogen and phosphorous concentrations. From one 30 L Niskin bottle, 25 L were gently transferred to a polycarbonate carboy fully wrapped in black plastic, using silicone tubing fitted with 200 µm mesh to eliminate mesozooplankton. At the same time, another 25 L from the other Niskin bottle were filtered through a Gelman SuporCap 100 capsule filter (0.2 µm) to obtain the water added to diluted treatments. Water filtered through the capsules showed undetectable Chl *a* concentrations and negligible numbers of fluorescent particles when they were examined by FCM. Unfiltered, prescreened seawater was gently mixed with filtered seawater in 2.3 L Nalgene polycarbonate containers to obtain two replicated dilutions with *f = *0.25, 0.50, and 0.75. Replicated dilutions with *f = *1 (with and without nutrient enrichment) were obtained by filling containers with unfiltered prescreened seawater. Initial Chl *a* concentration for each treatment were calculated as the product of the measured initial Chl *a* concentration at *f* = 1 and the different dilution factors.

The nutrient mixture added to the enriched treatments resulted in a final concentration of 1 µM ammonium (NH_4_Cl), 0.5 µM phosphate (H_3_PO_4_), 5 nM iron (FeSO_4_) and 0.1 nM manganese (MnSO_4_). Powder-free plastic gloves were used during all operations. Containers were kept in dark during the whole process until placed in on-deck incubators. Incubations started always 1.5 h after water collection and lasted approximately 21 h. Blue sheets of light filters were combined to simulate *in-situ* light intensity and spectra. When necessary, the combination of light filters was corrected after light measurements in the morning, thus correcting possible small shifts in the amount of irradiance reaching the different depths. Temperature was kept within ±0.5^o^C of *in situ* temperature by connecting a cooler and a heater to two thermostats. Water inside the incubator was homogenized by a submersible pump, which also shook gently the containers [20].

Samples taken at times *t_0_* and *t_f_* were also examined under *in vivo* FlowCam and a stereo microscope (Leica Z12.5) to check for potential damages to microzooplankton during sample gathering and handling, and during the incubations. FlowCam images showed undamaged microzooplankters, suggesting a reduced damage to microzooplankton during sample handling and during incubations. Samples processed by flow cytometry during EL were taken from each container at times *t_0_* and *t_f_*. We only show the results of dilution experiments for *Prochlorococcus* because of the generally low *R^2^* values and high standard errors of regressions obtained for the other groups. A solution of 1 µm fluorescent latex beads was added to each sample and used as a standard to estimate relative FL3 signals of *Prochlorococcus.* We used these data as proxies for chlorophyll fluorescence changes during the incubations, as these changes would affect Chl *a*-based growth rate estimates. Statistical analyses were conducted using Statistica 8 and R [31] softwares. Graphs were plotted using Grapher software and the R package *ggplot2* [32].

## Results

### Oceanographic Conditions

Temperature and salinity in WL were warmer and saltier than in EL, and the thermocline and halocline were deeper in EL (Fig. 2A, B, H, I). As a result, the upper mixed layer was also deeper in EL. The depth of the photic layer was around 105 m during both Lagrangian surveys. Nutrient concentrations were very low in the photic layer in both Lagrangians (NO_3_^−^ plus NO_2_^−^ <0.3 µM and PO_4_^−^ <20 nM). There was a sharp nutricline at 140 m depth in WL and at 130 m depth in EL (Fig. 2F, G, M, N). The DCM was located at 115 m and 110 m depth in WL and EL respectively (Fig. 2C, J). Average oxygen saturation was above 100% down to 108 m in WL and down to 113 m depth in EL (Fig. 2E and 2L). These levels of O_2_ saturation imply a net autotrophic balance since the last mixing event, when atmosphere and ocean O_2_ concentrations were equilibrated. Indeed, maximum values were found at 73 and 74 m depth in WL and EL, respectively. Oxygen concentration profiles followed similar patterns (Fig. 2D and 2K).

Temporal variation of physical-chemical variables was in general low within each Lagrangian survey, suggesting that we did indeed sample the same parcel of water over the 5 day survey (Fig 2, 3; see Aranguren et al. [21] for further details). A greater scatter of θ-*S* pairs was found between isotherms 22.5°C and 20.75°C in WL (located around 77 m and 137 m depth, respectively) and between isotherms 22.65°C and 19.85°C in EL (located around 77 m and 138 m depth, respectively) (Fig. 3). This was probably caused by turbulent mixing promoted by Kelvin-Helmholtz instability associated to internal waves, and by salt fingers (see Thorpe [33]).

### Plankton Abundance

**Figure 4:**
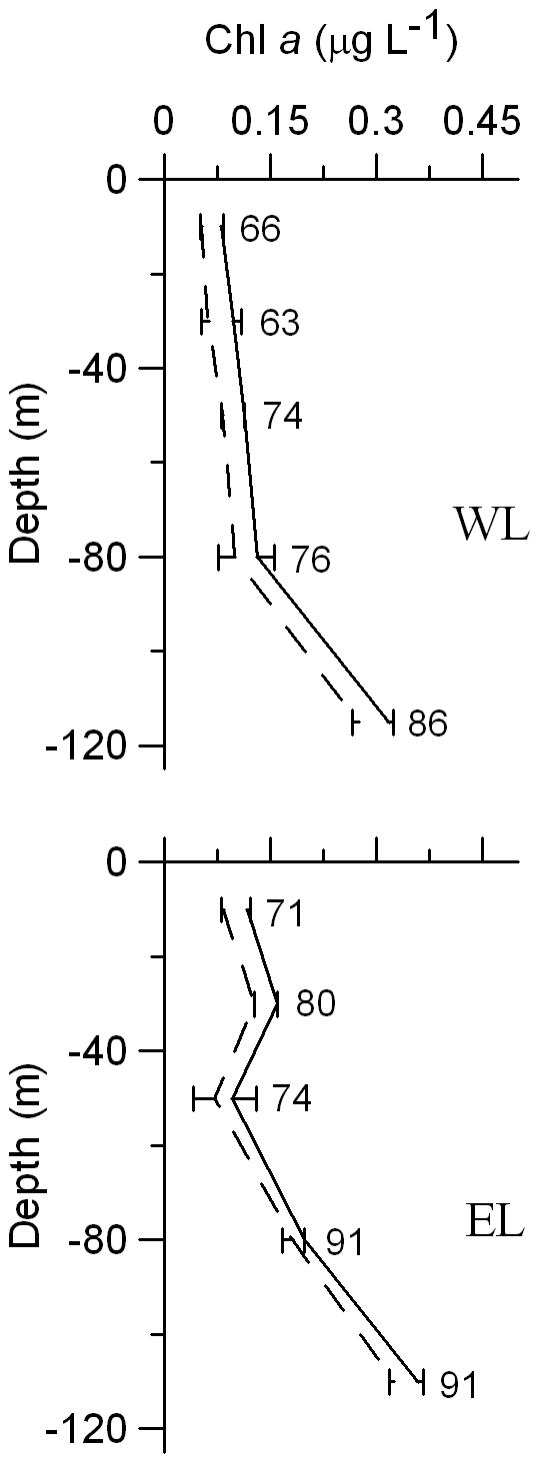
Vertical profiles of Chl *a* concentration up to the DCM during dilution experiments in WL and EL. Solid lines stand for total Chl *a* average values. Dashed lines indicate average Chl *a* <5 µm. Horizontal bars represent ± S.D. Numbers point out the percentage of Chl *a* <5 µm.

**Figure 5:**
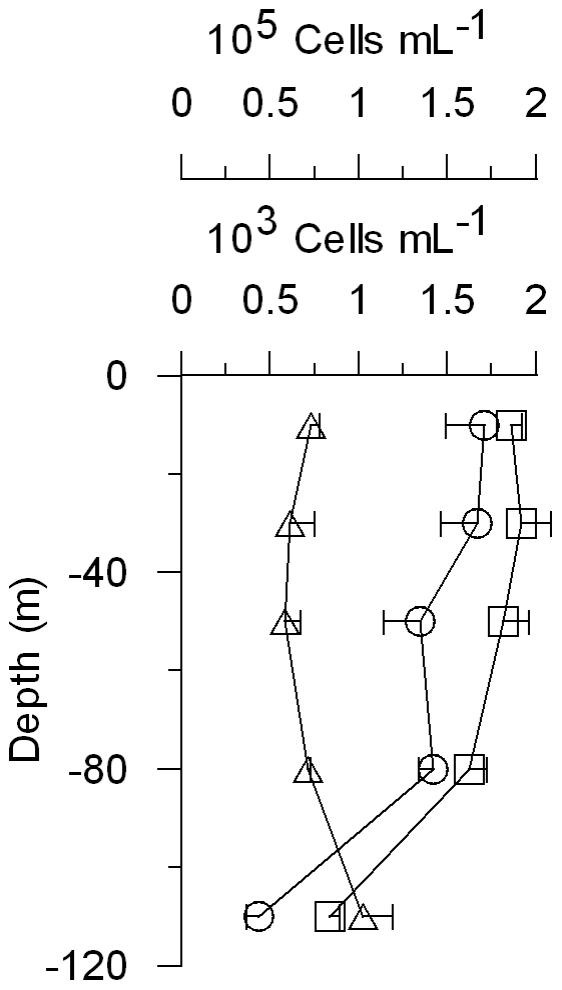
Picophytoplankton abundance during dilution experiments in EL. Vertical profiles of *Prochlorococcus* (rectangles), *Synechococcus* (circles) and picoeukaryotes (triangles) mean abundances up to the DCM. Symbols also point out sampling depths. Horizontal bars represent ± S.D. The 10^5^ cells mL^−1^ scale is for *Prochlorococcus*, whereas *Synechococcus* and picoekaryotes scale is 10^3^ cells mL^−1^.

Total integrated phytoplankton biomass was higher in EL than in WL, although if biomasses are expressed in C units values are similar (Table 1). Small phytoplankton integrated biomass was higher than the biomass of the large size fraction in both Lagrangian surveys (Table 1), although these differences diminish if biomasses are expressed in C units due to the higher C:Chl *a* ratios of the large size fraction. The contribution of small phytoplankton to total Chl *a* biomass was higher near the DCM (Fig. 4). The picophytoplankton community in EL was numerically dominated by *Prochlorococcus*, with abundances two orders of magnitude higher than *Synechococcus* and picoeukaryotes (Fig. 5). Both groups of cyanobacteria followed a similar depth distribution pattern, with lower abundances at the DCM. In contrast, picoeukaryotes were slightly more abundant at the DCM. *Prochlorococcus* was also dominant in terms of integrated C biomass (633 mg C m^−2^), followed by picoeukaryotes (139 mg C m^−2^) and *Synechococcus* (16 mg C m^−2^). Maximum abundances of diatoms, ciliates, radiolarians and copepod nauplii were found around 80 m depth in EL, where we also found a relative maximum abundance of dinoflagellates.

**Table 1:** Phytoplankton integrated biomass in the water column during our experiments.

Site	Size fraction (µm)	Biomass (mg Chl *a* m^−2^)	% Chl *a* <5	Biomass (mg C m^−2^)	% C <5
WL	< 5	15.19	78	801	52
	>5	4.23		724	
	total	19.42		1525	
EL	<5	20.83	87	970	65
	>5	3.26		531	
	total	24.09		1501	

Carbon based measurements were estimated using the C:Chl *a* ratios reported by Marañón et al. 2007 (see Methods). WL and EL refer to the West and East Lagrangian, respectively (see Figure 1). % Chl *a* <5: Contribution of phytoplankton Chl *a* biomass <5 µm to the total Chl *a* biomass. % C <5: Contribution of phytoplankton C biomass <5 µm to the total C biomass.

### Dilution Experiments

**Figure 6:**
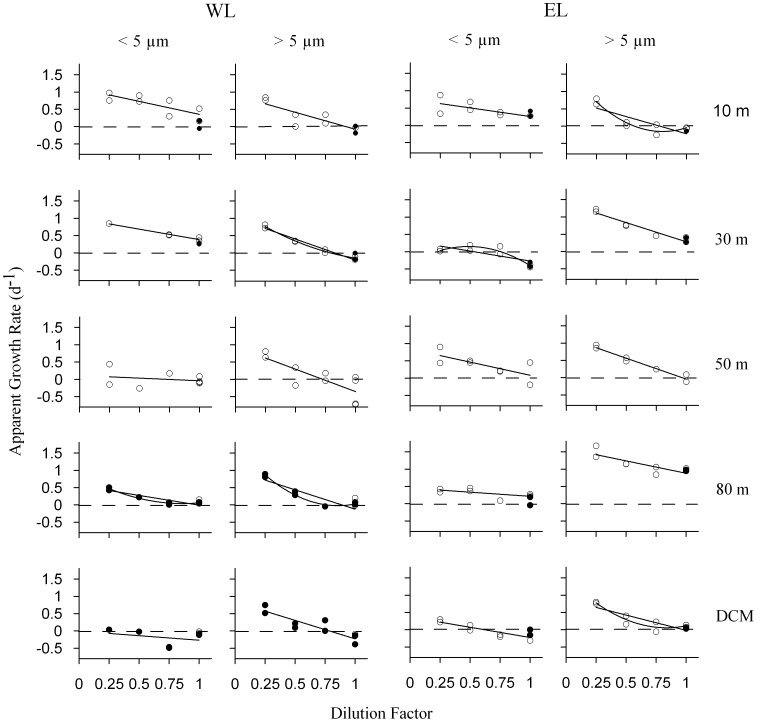
Plots of dilution experiments based on Chl *a* measurements. **Simple linear regressions between dilution factor and phytoplankton apparent growth rate for both phytoplankton size fractions and Lagrangians.** Quadratic fits are showed if they significantly (*p*<0.05) improve the relationship. White dots indicate phytoplankton apparent growth rate in treatments with no nutrients added. Black dots point out phytoplankton apparent growth rates in treatments with nutrient added. Dashed lines indicate apparent growth rate = 0.

Phytoplankton apparent growth rates increased linearly with the dilution factor in most of the experiments. However, in six experiments out of 20, the relationship significantly improved by fitting a quadratic function (*p*<0.05, see fig. 6), although explained variances by simple linear regression were quite high in all those cases (*R^2^*>0.53). Integrated phytoplankton growth and microzooplankton grazing rates obtained in both Lagrangian surveys were high and similar (Table 2), suggesting a similar global functioning of the ecosystem in the two areas of study. Nevertheless, rates in WL and EL were different at some depths (Table 3). Regarding the comparison between phytoplankton size fractions, integrated phytoplankton growth and microzooplankton grazing rates were lower for the small phytoplankton fraction (Table 2). Hence, rates of the small phytoplankton size fraction were similar or lower than the rates of the large fraction at all the depths analyzed (Table 3). Differences in growth rates between both phytoplankton size fractions were high below the UML in the two Lagrangian surveys, coinciding with the maximum phytoplankton growth rates detected for the large phytoplankton size fraction (Table 3).

**Table 2:** Integrated *μ*, *m* and *μ-m* balance (d^−1^) for both phytoplankton size fractions and Lagrangians.

Site	Size fraction (µm)	Int. *μ* (d^−1^)	Int. *m* (d^−1^)	Int. *μ-m* (d^−1^)	% Int. pNPP
WL	<5	0.57 (483)	0.44 (373)	0.13 (110)	77
	>5	1.04 (709)	1.17 (799)	−0.13 (−90)	113
	total	0.78 (1192)	0.77 (1172)	0.01 (20)	98
EL	<5	0.56 (529)	0.58 (557)	−0.02 (−28)	104
	>5	1.12 (614)	1.04 (568)	0.08 (46)	93
	total	0.76 (1143)	0.75 (1124)	0.01 (19)	98

In brackets besides each rate integral are their associated integrated pNPP, G and pNPP-G balances (mg C m^−2^ d^−1^). The final column (% Int. pNPP) indicates the percentage of phytoplankton grazed in the upper 125 m of the water column.

**Table 3:** Estimated growth and grazing rates for small (<5 µm) and large (>5 µm) phytoplankton size fractions.

Site	Depth (m)	Size fraction (µm)	*µ_n_* ± SE (d^−1^)	*μ_0_*± SE (d^−1^)	*μ_0_:µ_n_*	*m* ± SE (d^−1^)	r	*μ-m* balance (d^−1^)	% pNPP
WL	−10	<5	0.8	1.10±0.17	1.38	0.75±0.25	0.78*	0.35	68
	−10	>5	0.99	1.03±0.10	1.04	1.08±0.14	0.96**	−0.05	105
	−30	<5	0.87	0.99±0.05	1.14	0.60±0.07	0.99**	0.39	61
	−30	>5	1.12	1.01±0.07	0.9	1.22±0.10	0.98**	−0.21	121
	−50	<5		0.11±0.20		0.15±0.26	0.23	−0.04	136
	−50	>5		0.94±0.27		1.29±0.35	0.79**	−0.35	137
	−80	<5	0.56±0.08	0.66	1.18	0.56±0.12	0.91**	0.1	85
	−80	>5	1.00±0.16	1.21	1.21	1.12±0.23	0.89**	0.09	93
	−115	<5	0.00±0.28	0.22		0.26±0.37	0.33	−0.04	118
	−115	>5	0.84±0.16	0.95	1.13	1.07±0.23	0.89**	−0.12	113
EL	−10	<5	0.83	0.75±0.18	0.9	0.50±0.28	0.62	0.25	67
	−10	>5	0.8	0.77±0.20	0.96	0.99±0.29	0.81**	−0.22	129
	−30	<5	0.21	0.31±0.15	1.48	0.58±0.22	0.73*	−0.27	187
	−30	>5	1.45	1.39±0.09	0.96	1.11±0.13	0.97**	0.28	80
	−50	<5		0.84±0.20		0.76±0.30	0.72*	0.08	90
	−50	>5		1.17±0.07		1.21±0.10	0.98**	−0.04	103
	−80	<5	0.31	0.45±0.09	1.45	0.24±0.14	0.61	0.21	53
	−80	>5	1.68	1.60±0.14	0.95	0.72±0.20	0.82**	0.88	45
	−110	<5	0.64	0.45±0.05	0.7	0.80±0.09	0.97**	−0.35	178
	−110	>5	0.93	0.86±0.16	0.92	0.88±0.24	0.84**	−0.02	102

*µ_n_*: phytoplankton growth rate estimated from treatments with added nutrients. *µ_0_*: phytoplankton growth rate estimated from treatments with no nutrients added. *m*: microzooplankton grazing rate. *μ-m* balance: balance between *μ* and *m*. % pNPP: % Particulated net primary production grazed. SE: Standard error of regression parameters. **r significant at *p*<0.01. *r significant at *p*<0.05.

Phytoplankton growth was not nutrient-limited in the experiments. Median phytoplankton growth rates obtained in nutrient addition treatments were indistinguishable from median phytoplankton growth rates without added nutrients in the case of small (Wilcoxon matched pairs test, *p = *0.12, *n = *8) and large (Wilcoxon matched pairs test, *p* = 0.67, *n = *8) fractions. The ratios between phytoplankton growth rates without and with added nutrients (*μ_0:_ µ_n_*), were in general close to one (Table 3).

**Figure 7:**
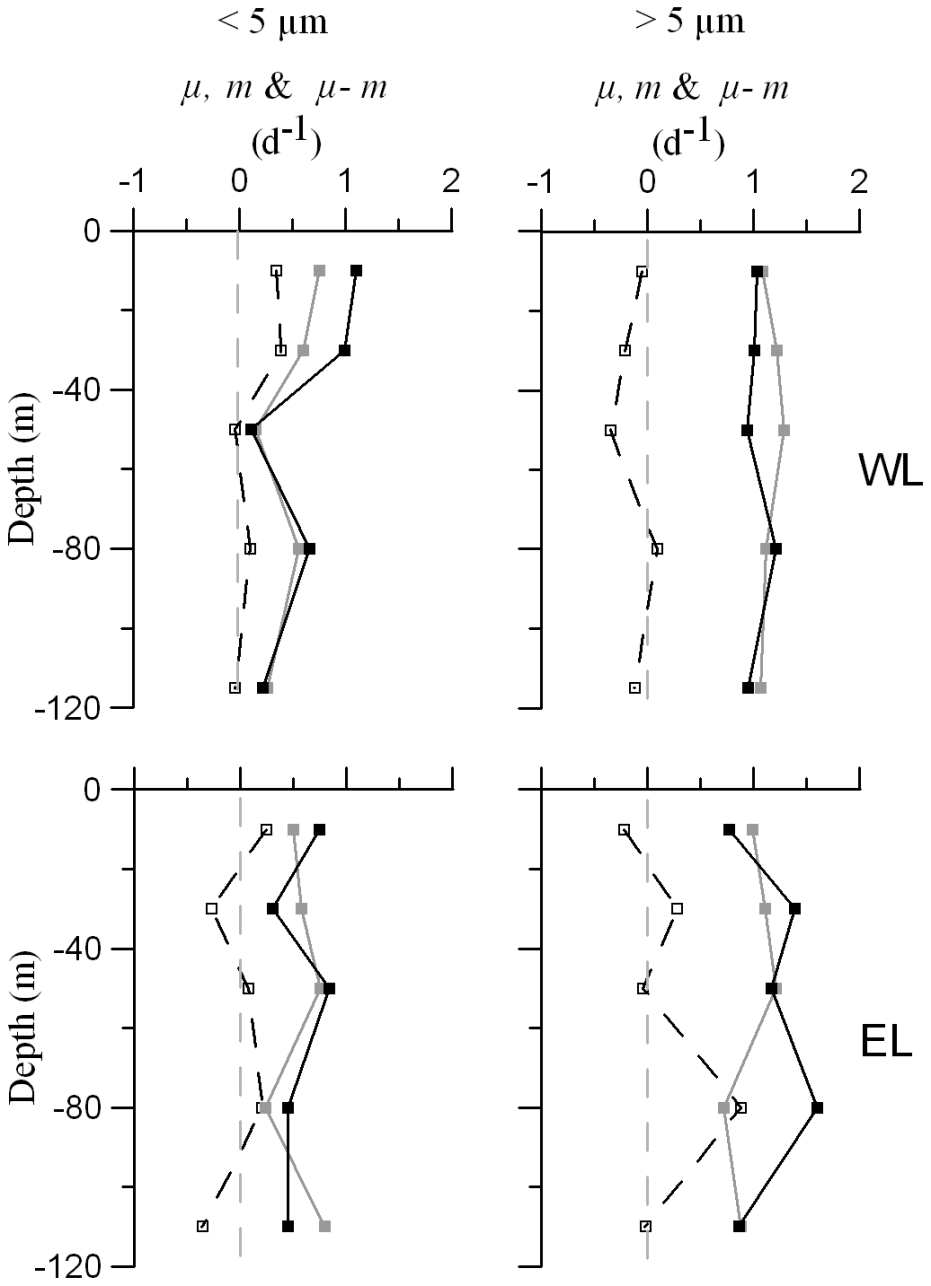
Vertical profiles summarizing the results of dilution experiments. Phytoplankton growth rates (black squares and lines), grazing rates (grey squares and lines) and *μ-m* balances (white squares and black dashed lines) of both phytoplankton size fractions and Lagrangians. Squares also point out the depths at which dilution experiments were performed. Grey dashed lines indicate the zero value.

We obtained a very good relationship between *μ-m* balances estimated from dilution experiments and net sea Chl *a* changes (data not shown; *R^2^* = 0.71, n = 6), providing further support to our experimental balances and rates. The total *μ-m* integrated balance was close to zero in WL (Table 2). The integrated balance for small phytoplankton was slightly positive, mainly due to the positive balance in the UML (Fig. 7). On the contrary, the integrated balance of large phytoplankton was slightly negative (Table 2), with positive values only around 80 m depth (Table 3, Fig. 7). The % pNPP consumed ranged between 61% and 136% for small phytoplankton and between 93% and 137% for large phytoplankton. Total *μ-m* integrated balance was also balanced in EL, being the *μ-m* integrated balances of both phytoplankton size fractions close to zero too (Table 2). The % pNPP grazed ranged between 53% and 187% for small phytoplantkon and between 45% and 129% in the case of the large fraction (Table 3). The *μ-m* balance was positive at 80 m depth, near the maximum % O_2_ saturation, in both Lagrangians and for both phytoplankton size fractions.

**Figure 8:**
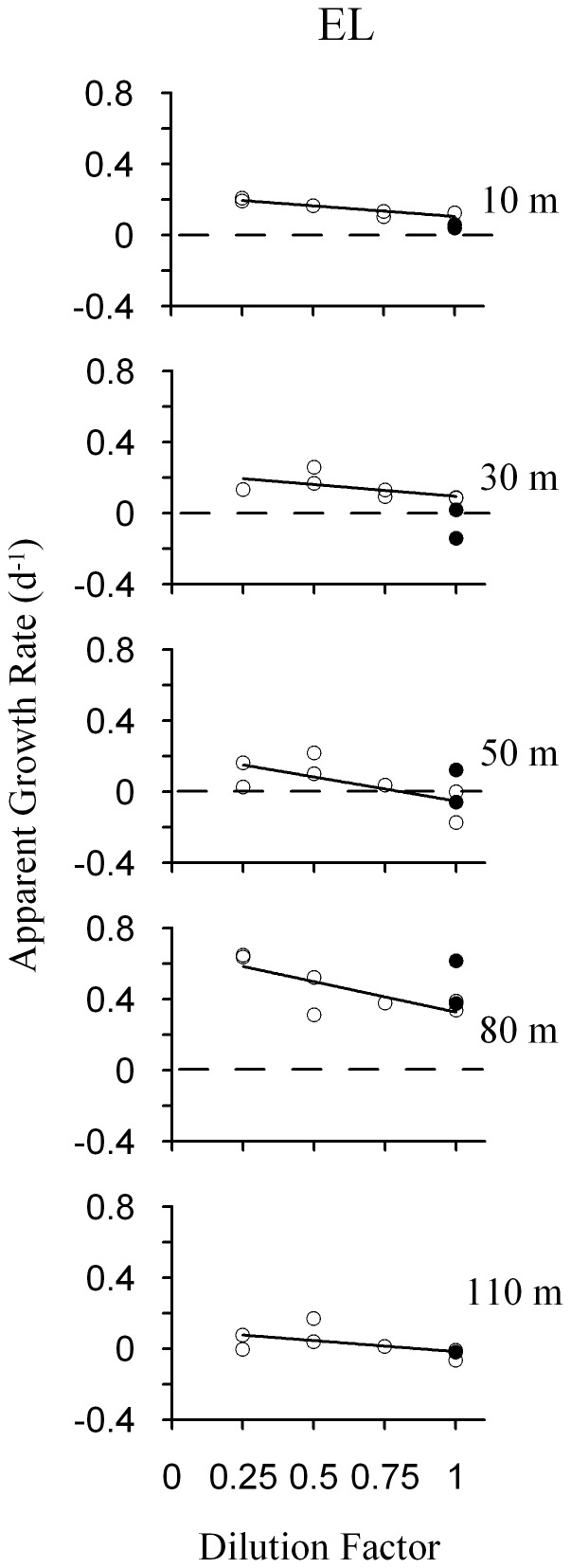
Plots of dilution experiments based on *Prochlorococcus* abundances. **Simple linear regressions between dilution factor and** ***Prochlorococcus*** **apparent growth rate in EL.** **** White dots: *Prochlorococcus* apparent growth rate in treatments without added nutrients. Black dots: *Prochlorococcus* apparent growth rates in nutrient added treatments. Dashed lines indicate apparent growth rate = 0.

*Prochlorococcus* growth rates at EL were nearly constant in the UML and relatively low (0.2 d−^1^), reaching maximum and lowest values at 80 m depth and DCM, respectively (Fig. 8; Table 4). Grazing rates were also maximum at 80 m depth; nevertheless they changed along the UML (Table 4). In all the experiments analyzed, relationship between dilution factor and *Prochlorococcus* apparent growth rate did not significantly improve by fitting a quadratic function (*p*>0.13). No differences between net growth rates of *Prochlorococcus* in treatments with and without nutrients were detected (Wilcoxon matched pairs test, *p* = 0.86, *n* = 9).

**Table 4:** Estimated growth and grazing rates for *Prochlorococcus* in EL.

Depth (m)	*µ_n_*(d^−1^)	*μ_0_*± SE(d^−1^)	*μ_0_:µ_n_*	*m* ± SE(d^−1^)	r	pN*P*P(10^3^ cells ml^−1^ d^−1^)	*P*G(10^3^ cells ml^−1^ d^−1^)	*μ-m* balance(d^−1^)	% *P*P 55
−10	0.17	0.22±0.02	1.29	0.12±0.03	0.90*	39	21.3	0.1	
−30	0.07	0.23±0.06	3.29	0.13±0.08	0.6	41.9	23.7	0.1	57
−50	0.3	0.22±0.09	0.73	0.27±0.13	0.68	35.5	43.6	−0.05	123
−80	0.83	0.67±0.08	0.81	0.34±0.12	0.78*	115.1	58.4	0.33	51
−110	0.07	0.12±0.06	1.71	0.1±0.09	0.53	9.2	7.7	0.02	83

*µ_n_*: *Prochlorococcus* growth rate estimated from treatments with added nutrients. *µ_0_*: *Prochlorococcus* growth rate estimated from treatments with no nutrients added. *m*: microzooplankton grazing rate. pN*P*P: Particulate net *Prochlorococcus* production. *P*G: *Prochlorococcus* grazing losses. *μ-m* balance: balance between *μ* and *m.* % *P*P: % *Prochlorococcus* production grazed. SE: Standard error of regression parameters. *r significant at *p*<0.05.

Initial and final relative FL3 signals of *Prochlorococcus* were different (Sign test, *p*<0.001, *n* = 39). Relative FL3 increased during incubations at all depths except at the DCM. Differences between initial and final relative SSC, an indicative of cell size, were also observed (Wilcoxon matched pairs test, *p* = 0.003, *n = *39) and followed a similar pattern as relative FL3 signal, although the magnitude of the changes was lower.

## Discussion

Dilution experiments were performed to assess ecosystem functioning and phytoplankton productivity in the eastern border and near the center of the North Atlantic Subtropical gyre. Despite the low nutrient concentrations, chlorophyll-based phytoplankton growth and grazing rates were high, suggesting a very dynamic ecosystem similar to the proposed by Goldman et al. [12]. In the following, we discuss the operation of the microbial community taking into account the high phytoplankton growth and grazing rates obtained. Also, we discuss the metabolic balance from *μ-m* balances and the O_2_ supersaturation found. In the first part of discussion following, we underline some of the caveats and uncertainties related to the methods employed.

### Potential Caveats of the Dilution Technique

The dilution technique is based on some assumptions [16] that must be validated to obtain correct estimates of phytoplankton growth and grazing rates. One of these assumptions is that dilution does not affect the estimates of both rates. For instance, several studies discuss the possibility that nutrient availabilities change across dilution treatments [16], [19]. Another possibility we are not able to reject is related to the potential effects of dilution on mixotrophy, especially considering their importance in oligotrophic environments [34]. Indeed, mixotrophs could increase their content of Chl *a* with dilution to obtain organic carbon by photosynthesis, compensating for the reduction of C obtained by predation (see Arenovski et al. [35] and references therein). This possible response would overestimate the rates obtained. The quantification of this effect remains a challenge for future dilution experiment studies in oligotrophic waters.

The observed increase in relative FL3 and SSC in *Prochlorococcus* cells seems to be the result of a light-dark cycle [36], although we can not entirely discard the occurrence of photoacclimation processes. The experiments lasted approximately 21 h, and they started at the end of the dark period, when a low percentage of cells are at the G2 phase (% G2), resulting in their FL3 and SSC relative signals being close to their daily minimum [37]. Then, it would be possible to detect an increase in those signals, especially if growth rates were high. Hence, we found a positive logarithmic correlation between *µ_0_* and the increase in the relative FL3 (*R^2^* = 0.99, *n* = 5) and SSC (*R^2^* = 0.67, *n* = 5) signals, despite *Prochlorococcus* growth rates would have to be low when photoacclimation occurs [38]. Therefore, the rise of those signals could mean the existence of a *Prochlorococcus* growth which was not detected. Thus, *Prochlorococcus* growth rates might be underestimated.

### Phytoplankton Growth and Microzooplankton Grazing Rates

The low nutrient concentrations found in both locations contrasted with the high phytoplankton growth rates obtained. They would be promoted by the high micro and nanozooplankton grazing rates detected [12], previously reported by Quevedo et al. [20] in surface waters and in the DCM. On one hand, grazers diminish phytoplankton biomass relaxing competition for nutrients. At the same time, grazers also take an active part in nutrient regeneration, increasing the amount of nutrients available to phytoplankton cells [14]. High phytoplankton growth rates would also be promoted by a variety of mechanisms known to diminish nutrient consumption or to improve nutrient uptake, like mixotrophy [34], phytoplankton associations [39], N_2_ fixation [40] and other biochemical and physiological mechanisms [41], [42]. Indeed we did not find differences in phytoplankton growth rates between treatments with and without nutrient enrichment, maybe because of the short incubation times. For all those reasons, the regulation of the system seems closer to a top-down control than to a bottom-up one during the time of study, although the low amount of nutrients available would influence phytoplankton community composition and its overall biomass.

Our integrated growth rates were within the range of values reported in other studies carried out in open ocean oligotrophic regions (e.g. [18], [43], [44]). Also, similar phytoplankton growth rates have been reported in the Subtropical Pacific using the ^14^C method [45], [46], although the rates reported here were greater than most of the ^14^C estimates reported in the NAST-E region [47], [48], [49]. Consequently, our pNPP estimates were also higher. However, the ^14^C method could largely underestimate primary production in oligotrophic environments [11], and, consequently, phytoplankton growth rates obtained with this methodology. This underestimation could be partially caused by the use of small incubation bottles [50], [51]. In addition, the high percentage of pNPP grazed, typical from tropical regions [9] and also observed in this study, could prevent the detection of a considerable amount of the fixed carbon. All these reasons make the existence of differences in production estimates between ^14 ^C method and other techniques possible (see Quay et al. [6]).

We obtained higher growth rates for the large phytoplankton fraction despite the assumed competitive advantage of small phytoplankton to exploit low nutrient concentrations waters [52]. Slightly higher metabolic rates for the large phytoplankton were also reached in these latitudes by using the ^14^C method [50], although similar [48], or even lower rates [49] were observed using the ^14^C method too. McCarthy and Goldman [53] suggested the existence of microscale nutrient patches resulting from zooplankton excretion and degradation of particulate organic matter. The existence of such nutrient patches, and the response of algal flagellates to them, was subsequently proved [54], [55], [56]. Large phytoplankton could take advantage of those patches because they generally have higher maximum nutrient uptake rates [57] and motilities [58]. In addition, the storage capacity and vertical migration of some groups of large phytoplankton like diatoms [59], dinoflagellates or cyanobacteria of the genus *Trichodesmium* [60], could provide an advantage to exploit the nutrient heterogeneity at the vertical scale too. In this way, we propose that the spatio-temporal variability of nutrient concentrations might explain the higher growth rates obtained for the large phytoplankton. As Polz et al. [61] proposed for bacteria, there could be two strategies followed by phytoplankton in oligotrophic environments: the passive oligotroph (“SS strategist” according to Reynoldśs scheme [58], “affinity adapted” in Sommeŕs [62] scheme), mainly adopted by small phytoplankton (e. g. *Prochlorococcus*), exploiting the low background nutrient concentrations; and the “opportuni-troph”, which might be the strategy adopted by most of the large phytoplankton fraction (e.g. some phytoflagellates or diatoms), exploiting the nutrient enriched environments at different spatiotemporal scales.

In general, the differences in growth between both fractions were higher below the UML, where large phytoplankton growth rates were the highest recorded. This observation was especially evident at 80 m depth, coinciding with the maximum O_2_ supersaturation. At 80 m depth in EL we found the maximum abundance of diatoms and a relative maximum of dinoflagellates too. The higher availability of nutrients resulting from turbulent mixing processes, together with vertical excursions to take up nutrients in enriched deeper waters [59], could result in higher growth rates for large phytoplankton, especially considering their higher photosynthetic efficiency under non-limiting conditions [63]. In addition, microzooplankton (ciliates, nauplii and metanauplii) abundances were also maxima at this layer in EL. These activities might enhance the formation of microscale nutrient patches and might consequently promote the advantage of “opportuni-trophs”, providing further enhancement to the very high growth rates detected for the large phytoplankton.

### Production-grazing Balances and Metabolic Balances

The percentage of pNPP grazed was at some depths far from 100%, especially in EL. This would indicate *μ-m* imbalances that might be related to predator-prey cycles. In both Lagrangian surveys, the depth around maximum O_2_ saturation seemed to be a net production zone of phytoplankton biomass (positive particulate *μ-m* balances at 80 m depth from Chl *a* analysis and *Prochlorococcus* counts), while the DCM was a net consumption zone (negative *μ-m* balances). However, without mixing events or other restoration processes, the *μ-m* imbalances would not persist for a long time: the high potential growth and grazing rates of protists [64] and the low carrying capacity of phytoplankton populations in oligotrophic subtropical gyres, would limit the duration of positive imbalances. At the same time, the low phytoplankton stocks would prevent long negative imbalances. According to this idea, the potential length and amplitude of the imbalances would be maximum in winter and early spring, when nutrient concentrations and phytoplankton carrying capacity is maximum, and they would attenuate in summer, when the strengthened stratification favors lower nutrient concentrations in the surface.

In spite of the imbalances detected for single depths, the integrated % pNPP grazed of small and large phytoplankton were close to 100%, except in the case of the small phytoplankton fraction in the WL. If both phytoplankton fractions are treated together, then the integrated % pNPPs were even closer to 100%, greater than the average 74.5% pNPP grazed reported for the open ocean [9]. Nevertheless, most dilution experiments analyzed in Calbet and Landry [9] were performed in the UML, where the % pNPP grazed is in general lower. Therefore, the coupled integrated *μ-m* balances for both Lagrangian surveys are the result of multiple compensated imbalances, increasing the coupling of the system with the size of the phytoplankton compartment.

The integrated *μ-m* balance reported could be approximated to a simultaneous metabolic balance (production- respiration) if i) grazing exerted by mesozooplankton is very low [65], [66], and mesozooplankton respiration is supported by carbon ingested from zooplankton consumption, ultimately coming from phytoplankton; and ii) bacterial respiration is fully supported by organic carbon coming from organisms of the contemporary community [67], *i.e.* eventually fixed by phytoplankton. Under these circumstances, even slightly negative integrated *μ-m* balances might imply autotrophic metabolic balances, given that part of the consumed phytoplankton would be finally exported and not respired in the upper water column. Therefore, according to the obtained *μ-m* balances, the water column analyzed would be slightly autotrophic during our experiments. Indeed, our results are within the confidence intervals reported to the net plankton metabolic balances during the same Lagragian surveys by using oxygen in vitro measurements [21]. On the other hand, assuming a 30% dark phytoplankton respiration of pNPP, and 18% of extracellular release (PER) of the carbon photoassimilated by phytoplankton [68], the integrated GPP would be at least 1.68 g C m^−2^ d^−1^ in the two study areas. This value is higher than the average community respiration found in the photic layer of the NAST-E province obtained from oxygen experiments [24], [69].

Regarding the metabolic balance during longer time periods, oxygen supersaturation up to around 110 m depth was observed during Lagrangian surveys and along the approximately 2600 Km transect carried out just before the Lagrangian surveys [70]. This implies an autotrophic metabolic balance too, although the observed O_2_ supersaturation could be partially caused by a temperature increase during spring and summer [71]. Other studies also reported oxygen saturation levels higher than 100% [24], [72]. Furthermore, net oxygen production and supersaturation was observed at other subtropical oligotrophic regions [6], [73], [74], especially in summer and fall [75].

Despite the *μ-m* balance and the high GPP and the O_2_ supersaturation found, most studies in the North Atlantic subtropical gyre report that net heterotrophic metabolic balance prevails [69], [72], [76]. These opposing situations could take place if net heterotrophic events were more common and longer than the autotrophic ones, which would be infrequent and brief, although they would allow O_2_ supersaturation [77]. However, the overestimation of respiration [78], [79], or the probable underestimation of primary production estimates based on O_2_ incubations [6], [79], [80], could produce the same results. If autotrophy is the common situation [74], subtropical gyres could be an important carbon sink.
